# Spatial distribution of canine visceral leishmaniasis and its relationship with human visceral leishmaniasis in a municipality of Rio de Janeiro, Brazil

**DOI:** 10.1590/0037-8682-0304-2025

**Published:** 2026-02-09

**Authors:** Anna Eduarda Oliveira Pires Gonçalves, José Ueleres Braga, Fernanda Nunes Santos, Sandro Antonio Pereira, Artur Augusto Velho Mendes, Lucas Keidel Oliveira, Adilson Benedito de Almeida, Rodrigo Caldas Menezes, Millena Borges Conti Fonseca, Elvira Maria Godinho Seixas Maciel

**Affiliations:** 1 Fundação Oswaldo Cruz, Escola Nacional de Saúde Pública Sérgio Arouca, Rio de Janeiro, RJ, Brasil.; 2 Fundação Oswaldo Cruz, Instituto Evandro Chagas, Laboratório de Pesquisa Clínica e Vigilância em Leishmanioses, Rio de Janeiro, RJ, Brasil.; 3 Fundação Oswaldo Cruz, Instituto Nacional de Infectologia Evandro Chagas, Laboratório de Pesquisa Clínica em Dermatozoonoses em Animais Domésticos, Rio de Janeiro, RJ, Brasil.; 4 Fundação Oswaldo Cruz, Instituto Carlos Chagas, Curitiba, PR, Brasil.; 5 Coordenadoria de Vigilância em Saúde Ambiental de Barra Mansa, Barra Mansa, RJ, Brasil.

**Keywords:** Visceral leishmaniasis, Spatial analysis, Public health surveillance

## Abstract

**Background:**

Visceral leishmaniasis (VL) is an infectious disease that represents a considerable public health problem. In Brazil, VL was gradually expanded in all the territory and from rural to urban areas. A notable concentration of VL emerged in 2010 in the Médio Paraíba region of the state of Rio de Janeiro, where the municipality of Barra Mansa is located. However, information on the geographic distribution of VL in this municipality is scarce. Therefore, this study aimed to characterize the spatial distribution of VL in Barra Mansa from 2014 to 2023.

**Methods:**

This exploratory epidemiological study involved creating point maps for human visceral leishmaniasis (HVL) and canine visceral leishmaniasis (CVL), as well as kernel density and cluster maps for CVL. In addition, maps were produced by superimposing the point maps of human cases with the cluster maps of canine infections.

**Results:**

In total, 99.1% of CVL-infected dogs and 100% of human cases were observed in the urban areas of the municipality. Persistent spatial clusters and hotspots were identified in urban areas adjacent to rural zones. Moreover, 55% of HVL cases were located within 600 m of a CVL spatial cluster. The closest clusters primarily formed up to 3 years before the HVL cases.

**Conclusion:**

These results suggest a relationship between urban-rural interface characteristics and high density of CVL. They also highlight the spatial and temporal closeness between CVL and HVL, corroborating the influence of these factors on the maintenance of the disease cycle.

## INTRODUCTION

Leishmaniasis is a major public health concern worldwide. It is an infectious, vector-borne disease caused by protozoan parasites of the *Leishmania* genus and transmitted between hosts through bites from female phlebotomine sandflies^1^. Visceral leishmaniasis (VL) in Brazil follows a zoonotic pattern, affecting not only humans but also domestic and wild animals^2^. From an epidemiological perspective, domestic dogs act as the main urban reservoirs of VL. Therefore, controlling the disease in dogs is a key focus of the national control program^3^.

VL has a wide global distribution. According to the World Health Organization, it has been reported in 83 countries or territories across all five continents^4^. In Latin America, 91% of cases in 2023 occurred in Brazil^5^. VL has recently been reported in all Brazilian regions, occurring in 24 of 27 Brazilian states^6^. This diffuse distribution pattern only emerged in the 1980s; prior to that, cases were primarily concentrated in the northeastern region before gradually spreading to other regions, particularly the southeastern region^7^ ^,^ although the northeastern region still reports the highest incidence rates^8^. These changes in VL distribution in the 1980s were not only restricted to geographic regions but also involved other epidemiological transformations. VL was previously restricted to rural areas but progressively began to occur in urban settings^9^. Although the number of nationally reported cases has been decreasing, endemic foci persist^8^.

The Southeast of Brazil experienced an increase in VL cases during the 1970s. The state of Rio de Janeiro (RJ) recorded the first case of human visceral leishmaniasis (HVL) in 1977, in the peri-urban area of the capital, Rio de Janeiro^10^. Since then, VL has spread to various regions within the state. Initially, cases were mostly concentrated in the metropolitan region; however, an important VL concentration was observed in the Médio Paraíba region by 2010^11^. The municipality of Barra Mansa, located in this region, registered its first case of HVL in 2011, resulting in the development of surveillance measures for the municipality, including entomological studies, active searches for infected dogs, and culling practices for seropositive animals^12^.

Although information about the spatial dynamics of VL is fundamental for understanding how the maintenance of the disease cycle occurs in territories, supporting the formulation and implementation of effective prevention and control strategies, and enabling effective allocation of resources to address the situation^13^ ^,^ ^14^, data on the spatial and temporal distribution of VL in space and time in the municipality of Barra Mansa remains scarce.

Given the tendency for VL cases to increase across the entire state of Rio de Janeiro, greater attention to the disease is required, and local territories need to be investigated to support the development of appropriate control measures^11^. Thus, this study aimed to describe the spatial distribution of canine visceral leishmaniasis (CVL) and evaluate its possible overlap with the spatial distribution of HVL cases in Barra Mansa (RJ) between 2014 and 2023. This will contribute to the understanding of VL dynamics and clarify the relationship between disease occurrence in dogs and humans, knowledge that remains considerably limited within the territorial context of the municipality of Barra Mansa (RJ), Brazil. 

## METHODS

### Study area and period

**FIGURE 1:**
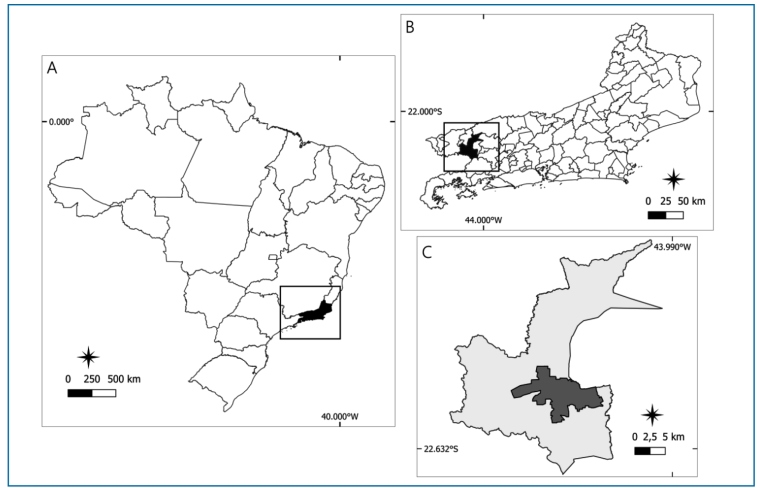
Location map of the study area; **A:** Brazil, highlighting the state of Rio de Janeiro; **B:** Rio de Janeiro state, highlighting the municipality of Barra Mansa; **C:** Barra Mansa municipality, distinguishing the rural (light gray) and urban (dark gray) zones.

This descriptive, cross-sectional exploratory epidemiological study was conducted between 2014 and 2023 in the municipality of Barra Mansa, located in the southern region of the state of Rio de Janeiro, Brazil (Figure 1).

### Study population and data collection

The study population consisted of domestic dogs diagnosed with CVL between 2014 and 2023, which were referred for culling to the Clinical Research Laboratory on Dermatozoonoses in Domestic Animals *(*Lapclin-Dermzoo) (*Laboratório de Pesquisa Clínica em Dermatozoonoses em Animais Domésticos*) at the Evandro Chagas National Institute of Infectious Diseases (INI), Oswaldo Cruz Foundation (Fiocruz), as well as HVL cases reported through SINAN during the same period. 

Data from the dog population were used, enabling the georeferencing of the animals’ residential locations. These data were obtained from documents from Lapclin-Dermzoo/INI/Fiocruz, which are completed on the day of the dogs’ culling procedure. Data on HVL cases also allowed for the georeferencing of patients’ residences, and these data were provided by the Barra Mansa Environmental Health Surveillance Department (Coordenadoria de Vigilância em Saúde Ambiental de Barra Mansa).

### Georeferencing

The residential addresses of animals with CVL and HVL cases were georeferenced using Map Maker (maps.co). Twelve places of residence were initially imprecisely georeferenced and subsequently manually corrected using Google Earth Pro (version 7.3.6.9)^15^. 

### Point maps

Point maps were created using QGIS (version 3.28.10)^16^ for exploratory purposes, based on the spatial data of both canines and humans with VL. The residences of culling dogs with CVL and HVL cases were represented as points on the maps. 

### Kernel density maps

Kernel density maps were generated using kernel estimation on the georeferenced addresses of animals subjected to culling owing to CVL, using QGIS (version 3.28.10)^16^. This analysis was used to identify hotspots, representing areas with a high concentration of canines infected with VL. 

### Detection of spatial clusters

The spatial clusters derived from grouping objects based on geographic similarity corresponded to areas where the studied event was concentrated in space^17^. In this study, spatial clusters were detected for dogs subjected to culling owing to CVL using the Density-Based Spatial Clustering of Applications with Noise (DBSCAN) technique. The analysis was conducted in R (version 4.3.4) using the DBSCAN package^18^. The resulting clusters were processed using QGIS (version 3.28.10)^16^ for improved visualization and enhanced analysis quality. 

### Location of human cases and spatial clusters of canine infection

Point maps of human cases were overlaid on the cluster maps of canine infections using QGIS (version 3.28.10)^16^ to analyze the possible relationship between the spatial distribution of CVL and HVL. This analysis enabled us to determine whether human cases were located inside or outside the cluster areas of infected canines and to measure the distance between HVL cases and CVL clusters. 

### Ethical considerations

This study was approved by the Research Ethics Committee of the National School of Public Health Sergio Arouca, Fiocruz, and the Research Ethics Committee of the INI/Fiocruz, under the Certificates of Ethical Appreciation Submission numbers 79468824.3.0000.5240 and 79468824.3.3001.5262, respectively. This study did not involve the manipulation of live animals, as only the addresses of dog residences were used to analyze the spatial distribution of infection. Thus, the project does not fall under the provisions of Brazilian Law No. 11.794/2008 and did not require approval from the Animal Ethics Committee.

## RESULTS

In total, 347 dogs from the municipality of Barra Mansa were registered and culled because of CVL at Lapclin-Dermzoo/INI/Fiocruz between 2010 and 2023. Twelve cases of HVL were reported in the municipality during this period; however, one case lacked a complete address, which made georeferencing impossible. Therefore, only 11 HVL cases were the analysis. From the point maps, 344 (99.1%) of the 347 infected dogs lived in urban areas of the municipality, whereas only three (0.9%) resided in rural areas. All 11 HVL cases were located in urban areas. Therefore, only georeferenced addresses in urban areas were used in the kernel density and cluster maps to improve visualization, and the three addresses of animals living in rural zones were excluded from the analysis. 

**FIGURE 2:**
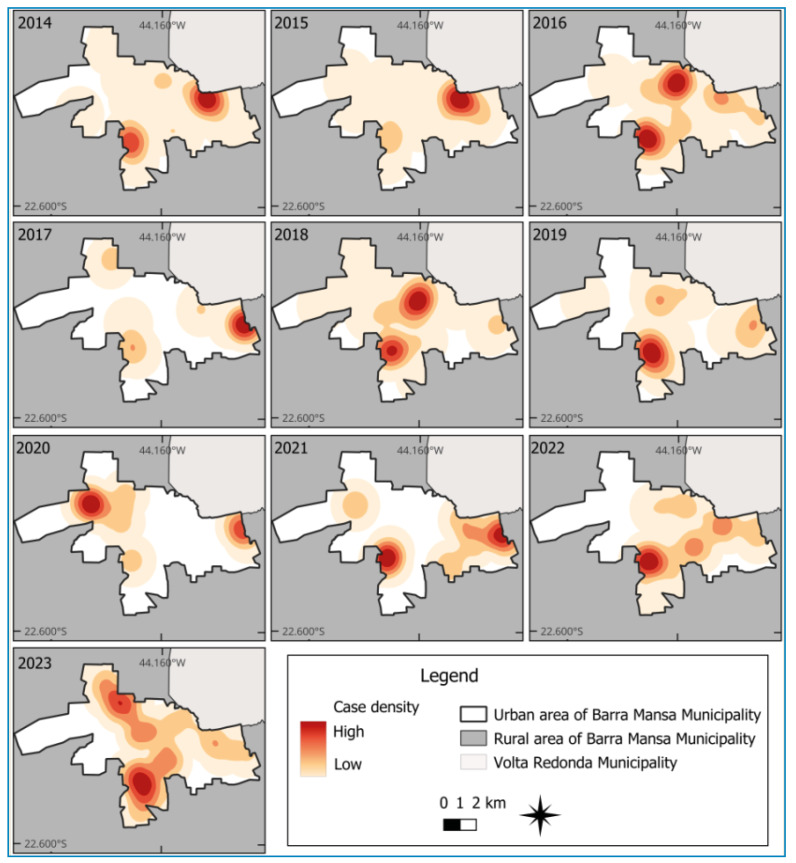
Kernel density maps showing the location of animals culled owing to CVL infection in the municipality of Barra Mansa (RJ), 2014‒2023.

The kernel density maps identified hotspots whose locations showed little variation between 2014 and 2023; five high-density areas were persistently observed: (i) near the urban-rural boundary of the municipality in the southwestern region, identified in all years of analysis, with stronger intensity in 2016, 2018, 2019, 2021, 2022, and 2023; (ii) near the boundary with the municipality of Volta Redonda in the northeastern region, observed in 2014, 2015, 2016, 2017, 2021, 2022, and 2023, with higher intensity in 2014 and 2015; (iii) in the northeastern region, persistent in 2014, 2016, 2018, 2019, 2022, and 2023, with higher intensity observed in 2016 and 2018; (iv) in the eastern region, in the transition zone between urban and rural areas near the boundary with the municipality of Volta Redonda, observed in 2017, 2018, 2019, 2020, and 2021, with higher intensity in 2017, 2020, and 2021; (v) near the boundary between the urban and rural areas in the northwest region of the municipality, identified in 2017 and 2023 (Figure 2).

**FIGURE 3:**
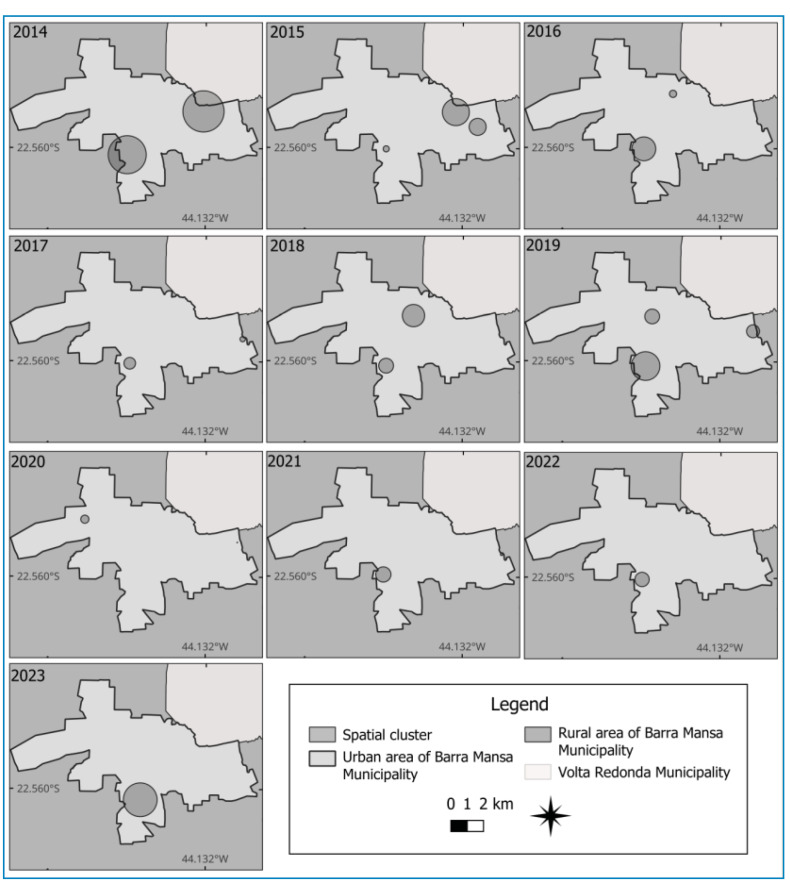
Cluster maps showing the locations of animals culled owing to CVL infection in the municipality of Barra Mansa (RJ), Brazil, 2014‒2023.

In the spatial cluster analysis of dogs culled for CVL, one cluster was identified near the urban-rural boundary in the southwestern region for each year of analysis, except in 2020, with the largest clusters observed in 2014, 2016, 2019, and 2023. In addition to this cluster, which was found persistently, another cluster near the boundary with the municipality of Volta Redonda in the northeastern region was identified in the years 2014 and 2015. In addition to these, smaller clusters were persistently formed in the years 2017, 2019, and 2020 in the urban-rural transition zone of the eastern region, near the boundary with the municipality of Volta Redonda. Finally, another cluster also persisted in the northeastern region between 2018 and 2019 (Figure 3). 

**FIGURE 4:**
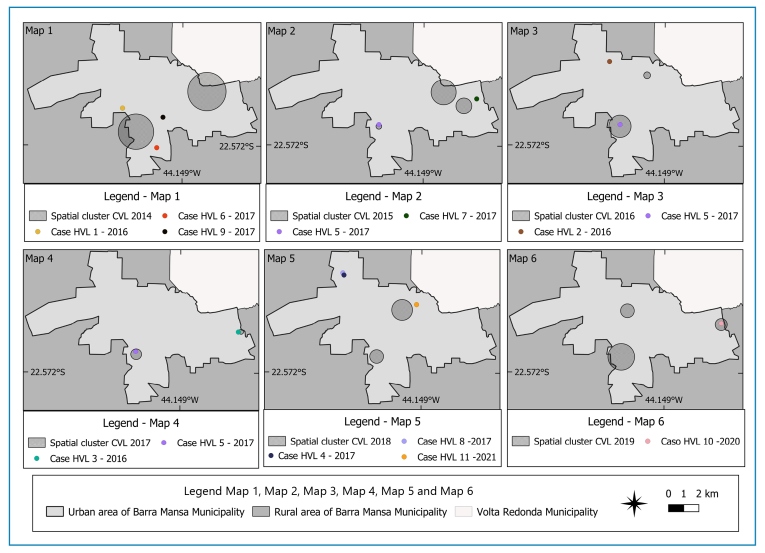
Cluster maps of dogs infected with CVL overlaid with the point map of HVL cases in the municipality of Barra Mansa (RJ), Brazil, between 2014 and 2023.

By overlaying the point map of human case residences onto the cluster map of dogs culled for CVL each year, the spatial cluster closest to each HVL case was identified. The observations were as follows: (i) from the spatial CVL clusters formed in 2014, one HVL case from 2016 and two from 2017 were closest; (ii) from the spatial CVL clusters formed in 2015, two HVL cases from 2017 were closest; (iii) from the spatial CVL clusters formed in 2016, one HVL case from 2016 and one from 2017 were closest; (iv) from the spatial clusters formed in 2017, one HVL case from 2016 and one from 2017 were closest; (v) from the spatial clusters formed in 2018, two HVL cases were from 2017 and one from 2021 ware closest; and (vi) from one of the spatial CVL clusters formed in 2019, the closest HVL case was from 2020 (Figure 4). 

Analysis of the distances between the residences of HVL cases and canine spatial clusters revealed that 55% of human cases were located within 600 m of an infectious canine spatial cluster. In addition, 72.7% of human cases were within 1,200 m of such clusters (Table 1).

**TABLE 1:** Frequency of HVL cases based on their minimum distances to canine VL infection clusters in Barra Mansa (RJ), Brazil, 2014‒2023.

Distance	n	%	% Cumulative
0 m‒600 m	6	55%	55%
601 m‒1200 m	2	18.2%	72.7%
1201 m‒2400 m	0	0%	72.7%
>2400 m	3	27.3%	100%
**Total**	**11**	**100%**	**100 %**

## DISCUSSION

This study aimed to describe the distribution of VL in Barra Mansa municipality between 2014 and 2023, leading to important findings. The point maps showing the residences of dogs culled owing to CVL in the municipality of Barra Mansa indicated that 99.1% of the animals lived in urban areas. Furthermore, point maps showing the locations of HVL residences indicated that all individuals (100%) lived within the urban perimeter. These findings corroborate the descriptions from a 2014 study on the epidemiological transition pattern of VL, which, beginning in the 1980s, shifted from predominantly rural areas to progressively urban locations^9^. Previous studies have reported higher circulation of HVL and CVL in urban areas than in rural ones^19^ ^,^ ^20^ *.*

However, rural populations often have less access to health than urban populations^21^. Therefore, barriers to identifying and reporting health problems in rural areas may result in fewer disease notifications. In addition, since epidemiological canine surveys in the Barra Mansa municipality were conducted following human or canine cases, the identification of infected animals may differ as extreme sensitivity to animals’ needs and care is frequently practiced in urban contexts^22^. This can contribute to greater attention to dog health by their owners, facilitating the identification of CVL cases. These identified cases can then serve as starting points for epidemiological investigations. Therefore, the detection and reporting of CVL cases may differ between urban and rural areas. 

The concentration of dogs subjected to culling owing to CVL, as observed in the kernel density maps, has persisted over the years in certain areas, maintaining hotspots. However, increases in density were also observed in these regions during some periods. High disease transmission in these territories may explain the presence of these hotspots. Notably, this study is a descriptive and exploratory investigation, using case counts as the basis for analysis without considering the size of the study population. Thus, the high concentration of cases in these territories may be associated with the high density of dog populations, since the absolute number of infected animals tends to be greater even if the infection rate in larger populations is the same. Areas of higher density may reflect increased diagnoses resulting from more targeted surveillance in territories where the disease is already established, which can contribute to the persistence or intensification of hotspots. 

Based on the cluster maps, spatial clusters of animals subjected to culling because of CVL were persistently observed in two locations within urban areas bordering rural zones: one located in the southwestern region and the other in the eastern region. Similarly, hotspots in kernel density maps also persisted in these regions. These findings are consistent with those of a previous study that showed a high density of CVL cases in peripheral neighborhoods located in urban-rural transition areas^20^. As this was an exploratory study, the results suggest that these patterns may be related to characteristics often present in urban-rural border neighborhoods, such as residual vegetation due to deforestation, abrupt population settlement, and unplanned land use, which may be directly associated with high incidences of VL^23^. Thus, it is hypothesized that the environmental characteristics frequently observed in peri-urban areas may be associated with the high occurrence of CVL-infected animals in the municipality of Barra Mansa, as has also been reported in other regions.

The findings of this study indicate the spatial proximity between human HVL cases and CVL-infected dogs during the study period in the municipality. Slightly over half of the HVL cases occurred within 600 m of a canine spatial cluster. It is important to note that sandflies can reach distances greater than 1000 m, as demonstrated in a study conducted in France^24^. However, research carried out in Brazil indicated a maximum flight range of 250 m, with most insects reaching distances of up to 100 m^25^. This finding is consistent with the results of a study conducted in 2018^19^ in the municipality of Ipatinga, Minas Gerais (MG), which suggested a relationship between HVL and CVL based on visual inspection. Although this study is exploratory in nature, the findings corroborate the results of other studies that indicate an association between areas with higher CVL infection and HVL cases^26^ ^-^ ^28^. 

Areas with HVL cases are the focus of surveillance activities in the municipality of Barra Mansa. Therefore, such an approach may increase the number of diagnosed canines due to intensified serological surveys in these territories^19^. However, temporal proximity was also identified when evaluating the spatial proximity between the detected residence clusters of dogs subjected to culling because of CVL and the nearest HVL cases. In most cases, the closest clusters occurred up to 3 years before the HVL case, with the exception of three HVL cases, which were closer to clusters formed by the residences of dogs subjected to culling owing to CVL 1 year after the human case. Despite these exceptions, the findings of this study are similar to those described in previous studies in which spatial clusters of infected dogs were recorded 6 years before the emergence of the HVL cluster^29^. This also corroborates a study conducted in 2001in the municipality of Belo Horizonte (MG), where canine infections preceded human infection cases^30^.

The results of this study support the influence of environmental and temporal factors on the maintenance of the VL transmission cycle. This pattern has been observed in Brazil since the 1980s. The present study found that most of the animals subjected to culling owing to CVL and total HVL cases in Barra Mansa (RJ) between 2014 and 2023 resided within the urban perimeter of the municipality. Both spatial clusters and hotspots of culled dogs were mainly and persistently located in urban areas near the urban-rural transition. This suggests a relationship between high CVL density and peripheral or peri-urban neighborhoods in the municipality, which are characterized by deforestation, unplanned occupation, and abrupt population growth. In addition, over half of the HVL cases were located within 600 m of a canine spatial cluster, indicating that the distribution of the disease in animals and humans may be related. Furthermore, the clusters closest to HVL cases were mostly formed by animals subjected to culling because of CVL prior to the human cases, highlighting a temporal relationship consistent with findings reported in the literature. 

## Data Availability

Data-not-available.
